# The efficacy of cognitive behavioral therapy for insomnia in adolescents: a systematic review and meta-analysis of randomized controlled trials

**DOI:** 10.3389/fpubh.2024.1413694

**Published:** 2024-11-19

**Authors:** Zhengyang Mei, Chenyi Cai, Shulai Luo, Yuanzhuo Zhang, Chifong Lam, Shi Luo

**Affiliations:** ^1^School of Physical Education, Southwest University, Chongqing, China; ^2^Key Laboratory of Cognition and Personality, Faculty of Psychology, Ministry of Education, Southwest University, Chongqing, China

**Keywords:** adolescents, cognitive behavioral therapy, insomnia, sleep, meta-analysis

## Abstract

**Objective:**

The objective of this systematic review and meta-analysis was to evaluate the overall efficacy of cognitive behavioral therapy for insomnia (CBT-I) in treating insomnia in adolescents, and to examine the efficacy of CBT-I on different sleep-related outcomes in this population.

**Methods:**

Randomized controlled trials (RCTs) of CBT-I on insomnia in adolescents were identified using electronic databases and manual searches. The Revised Cochrane risk-of-bias tool for randomized trials (RoB 2) was used to assess risk of bias in RCTs. A standardized mean difference (SMD) with a 95% confidence interval (CI) was used to combine effect sizes. A sensitivity analysis was performed for each outcome using a stepwise elimination method to assess whether the pooled results were significantly affected by individual studies.

**Results:**

The analysis included 8 RCTs involving a total of 599 participants. The meta-analysis indicated that marked and statistically significant improvements in insomnia (SMD = −1.06; 95% CI -1.65 to −0.47; *p* < 0.01), sleep onset latency (SMD = −0.99; 95% CI -1.65 to −0.32; *p* < 0.01), total sleep time (SMD = 0.50; 95% CI 0.10 to 0.90; *p* = 0.01), and sleep efficiency (SMD = 0.57; 95% CI 0.26 to 0.87; *p* < 0.01) were observed at post-treatment time point following CBT-I. At follow-up time point, a statistically significant improvement in insomnia (SMD = −0.79; 95% CI -1.42 to −0.17; *p* = 0.01) was observed following CBT-I.

**Conclusion:**

CBT-I was effective in improving insomnia in adolescents and some sleep-related outcomes, including sleep onset latency, total sleep time, and sleep efficiency. CBT-I was characterized by low risk and high therapeutic benefits and could serve as alternative or adjuvant approaches to medication for the treatment of insomnia. Considering the advantages in terms of safety and efficacy, CBT-I should be the preferred intervention for the treatment of insomnia in adolescents.

**Systematic Review Registration:**

https://www.crd.york.ac.uk/prospero/, CRD42024526102.

## 1 Introduction

Insomnia refers to a sleep disorder caused by physical, psychological and social stressors, usually accompanied by non-adaptive behaviors and hyperarousal, leading to the subjective experience of disturbed sleep (1). Worldwide, insomnia is the most common sleep disorder among adolescents, with a prevalence rate ranging from 7.8% to 23.8% (2–5). In addition, according to the White Paper on Sleep Health of Chinese Residents 2024, more than half of adolescents used to fall asleep after midnight, and accompanied by difficulties in falling asleep, restless sleep and nocturia, with insomnia being a major concern. Insomnia is predominantly characterized by dissatisfaction with sleep duration or quality and difficulties initiating or maintaining sleep, along with substantial distress and impairments of daytime functioning (6). Based on the Diagnostic and Statistical Manual of Mental Disorders 5 (DSM-V), diagnosis of insomnia is made when sleep difficulties are present for ≥3 nights per week and last for >3 months (7). Adolescence is not only a critical stage for changes in their sleep needs (8), but also a vulnerable period for the development of insomnia (9). During this period, adolescents are often exposed to various stressors, including academic stress (2), parent-adolescent relationship (10), parental conflicts (11), parental psychological control (12), and parenting styles (13). The cumulative effect of these stressors may increase the risk of insomnia in adolescents. There is a vast amount of evidence that insomnia is prevalent among adolescents (2, 3, 14–17), and that insomnia is associated with a variety of problematic behaviors and psychiatric disorders, including suicidal tendency (18), substance abuse (19), mobile phone use addiction (20), and symptoms of depression and anxiety (21, 22). These facts underline that insomnia not only severely affects adolescents’ physical and mental health, but also places a heavy burden on society and family.

Several countermeasures have been developed to help adolescents cope successfully with stress and improve insomnia, including Benzodiazepines (23), Ramelteon (24), Melatonin (25), and Suvorexant (26), which have been proved to be effective in treating insomnia. However, the prolonged use of such drugs can result in several side effects, such as long-term dependence, tolerance, and even rebound insomnia upon discontinuation (27–29). In this context, cognitive behavioral therapy for insomnia (CBT-I) may help to address these limitations. CBT-I is a psychotherapy focused on improving sleep quality and designing to guide patients to modify their behavioral and thinking patterns (6). Common CBT-I includes cognitive therapy, stimulus control therapy, sleep restriction therapy, sleep hygiene education, and relaxation therapy (6, 30–32). The feasibility and efficacy of CBT-I in treating insomnia in adolescents have been supported by numerous evidence (33–37). For instance, sleep hygiene education has been found to be effective in reducing sleep onset latency, regulating sleep cycles, and improving the duration of insomnia in adolescents (38, 39). Relaxation therapy is thought to have beneficial effects on wake time after sleep onset, sleep efficiency and quality, and can improve poor sleep symptoms in adolescents (40).

In terms of evaluating the overall efficacy of CBT-I in treating insomnia in adolescents, it can be assessed directly using the Insomnia Severity Index (ISI) and Holland Sleep Disorder Questionnaire (HSDQ), and indirectly through measuring different sleep-related outcomes, including sleep onset latency (SOL), total sleep time (TST), and sleep efficiency (SE). However, the scales to evaluate insomnia and sleep-related outcomes in published randomized controlled trials (RCTs) on CBT-I that improves insomnia in adolescents vary, resulting in different effect sizes. For adolescents exposed to many stressors, treating insomnia with CBT-I rather than medication may help them improve their quality of life (41) and some of the psychiatric disorders (e.g., depression, anxiety) that are highly associated with insomnia (36, 42), thereby mitigating the potential adverse effects caused by stressful situations. The objective of this systematic review and meta-analysis was to evaluate the overall efficacy of CBT-I in treating insomnia in adolescents, and to examine the efficacy of CBT-I on different sleep-related outcomes in this population.

## 2 Methods

This systematic review and meta-analysis followed the Preferred Reporting Items for Systematic Reviews and Meta-Analyses (PRISMA 2020) and was registered in the International Prospective Register of Systematic Reviews (PROSPERO), under number CRD42024526102.

### 2.1 Search strategy

Based on medical subject headings and free-text terms, a search was conducted across six databases: PubMed, Embase, EBSCOhost, Scopus, Web of Science, and APA PsycINFO. Additionally, the Google database was manually searched for relevant studies. The search timeframe was from the inception of each database to March 2024. The search strategy is presented in Table 1, per the PubMed database.

**Table 1:** PubMed search strategy.

#1	Insomnia [MeSH Terms]
#2	Insomnia [Title/Abstract] OR Insomnias [Title/Abstract] OR Sleep quality [Title/Abstract] OR Sleeplessness [Title/Abstract] OR Sleep disorder [Title/Abstract] OR Sleep disorders [Title/Abstract] OR Sleep disturbance [Title/Abstract] OR Sleep disturbances [Title/Abstract] OR Sleep Initiation Dysfunction [Title/Abstract] OR Sleep Initiation Dysfunctions [Title/Abstract] OR Dysfunction, Sleep Initiation [Title/Abstract] OR Dysfunctions, Sleep Initiation [Title/Abstract]
#3	#1 OR #2
#4	Cognitive behavio* therapy [Title/Abstract] OR Cognitive behavio* therapies [Title/Abstract] OR CBT [Title/Abstract] OR CBTI [Title/Abstract] OR Cognitive therapy [Title/Abstract] OR Cognitive therapies [Title/Abstract] OR Behavio* therapy [Title/Abstract] OR Behavio* therapies [Title/Abstract] OR Stimulus control [Title/Abstract] OR Sleep restriction [Title/Abstract] OR Sleep hygiene [Title/Abstract] OR Relaxation [Title/Abstract] OR psychotherapy [Title/Abstract]
#5	Adolescent [Title/Abstract] OR Adolescents [Title/Abstract] OR Adolescence [Title/Abstract] OR Teens [Title/Abstract] OR Teen [Title/Abstract] OR Teenagers [Title/Abstract] OR Teenager [Title/Abstract] OR Youths [Title/Abstract] OR Youth [Title/Abstract]
#6	Randomized controlled trial [Publication Type] OR Randomized [Title/Abstract] OR Placebo [Title/Abstract]
#7	#3 AND #4 AND #5 AND #6

The asterisk (*) was used as a wildcard to represent one or more characters, allowing for the retrieval of all variations of a root word.

### 2.2 Inclusion and exclusion criteria

The inclusion and exclusion criteria followed the PICOS principle. Specifically, studies were included in the present systematic review and meta-analysis if they met the following criteria: (P) population: adolescent and youth groups (ages between 6 and 19 years); (I) intervention: CBT-I was used as the generic term for interventions including cognitive behavioral therapy, stimulus control therapy, sleep restriction therapy, sleep hygiene education, and relaxation therapy; (C) control: control group received only routine treatment or appropriate rehabilitation intervention; (O) outcome: any assessment for insomnia or sleep-related outcomes; and (S) study design: randomized controlled trials.

Studies were excluded in the present systematic review and meta-analysis if they met the following criteria: (P) population: not adolescents; (I) intervention: interventions that were not CBT-I; (C) control: inappropriate control conditions; (O) outcome: studies that did not assess insomnia or sleep-related outcomes; and (S) study design: non-randomized controlled trials, such as quasi-experiments, study protocols, review, conference proceedings, comments, etc.

### 2.3 Study selection and quality assessment

According to the predetermined inclusion and exclusion criteria, two independent researchers (ZYM and CYC) used EndNote 20.6 bibliographic software for evidence selection. Duplicates were excluded when the references were imported into EndNote 20.6 and the remaining duplicates were manually removed. Two independent researchers screened and checked the references based on information such as the title, abstract, and full text. During the study selection process, any controversies were discussed and addressed by consulting the third author (SL).

The Revised Cochrane risk-of-bias tool for randomized trials (RoB 2) was used to assess the risk of bias in RCTs, in the following five respects: (1) randomization process, (2) deviations from intended interventions, (3) missing outcome data, (4) measurement of the outcome, and (5) selection of the reported result. For each eligible study, the overall risk of bias was assessed as either low risk of bias, with some concerns, or high risk of bias. During the quality assessment process, any controversies were discussed and addressed by consulting the third author (SL).

### 2.4 Data extraction

Using a data extraction form that included relevant information, two independent researchers collected the following data from each included study: (1) basic information, including the first author, country, and year of publication, (2) participant characteristics, including mean (standard deviations) age, sample size, and percentage of boys, (3) diagnostic criteria of insomnia, (4) intervention and control, and (5) sleep-related outcomes.

### 2.5 Statistical analysis

All the outcomes of this systematic review and meta-analysis were insomnia, sleep onset latency (SOL), total sleep time (TST), and sleep efficiency (SE). The endpoints for the outcomes were assessed at two time points, which were defined for this analysis: immediate post-treatment and follow-up (1 month to 6 months after completion of the intervention). As there were differences in the measurement tools used in the different RCTs, the standardized mean difference (SMD) with a 95% confidence interval (CI) was used to combine effect sizes (43). For all meta-analyses, heterogeneity among studies was assessed using the Chi-square test based on *Q*-test and *I^2^* statistics with a significance level of *p*-value <0.05 (44). According to the recommendations of Cochran’s handbook, when *p*-value <0.10 or *I^2^* > 50%, there was a significant heterogeneity, and a random-effect model was used to merge the results. Otherwise, a fixed-effect model was used to merge the results when there was no significant heterogeneity (*p*-value >0.10 or *I^2^* < 50%) (43). Since the number of trials included was less than 10, the publication bias was not assessed. A sensitivity analysis was performed for each outcome using a stepwise elimination method to assess whether the pooled results were significantly affected by individual studies (43). All statistical analyses of this study were performed using Stata 18.0 software.

## 3 Results

### 3.1 Literature search and eligible studies

**Figure 1:**
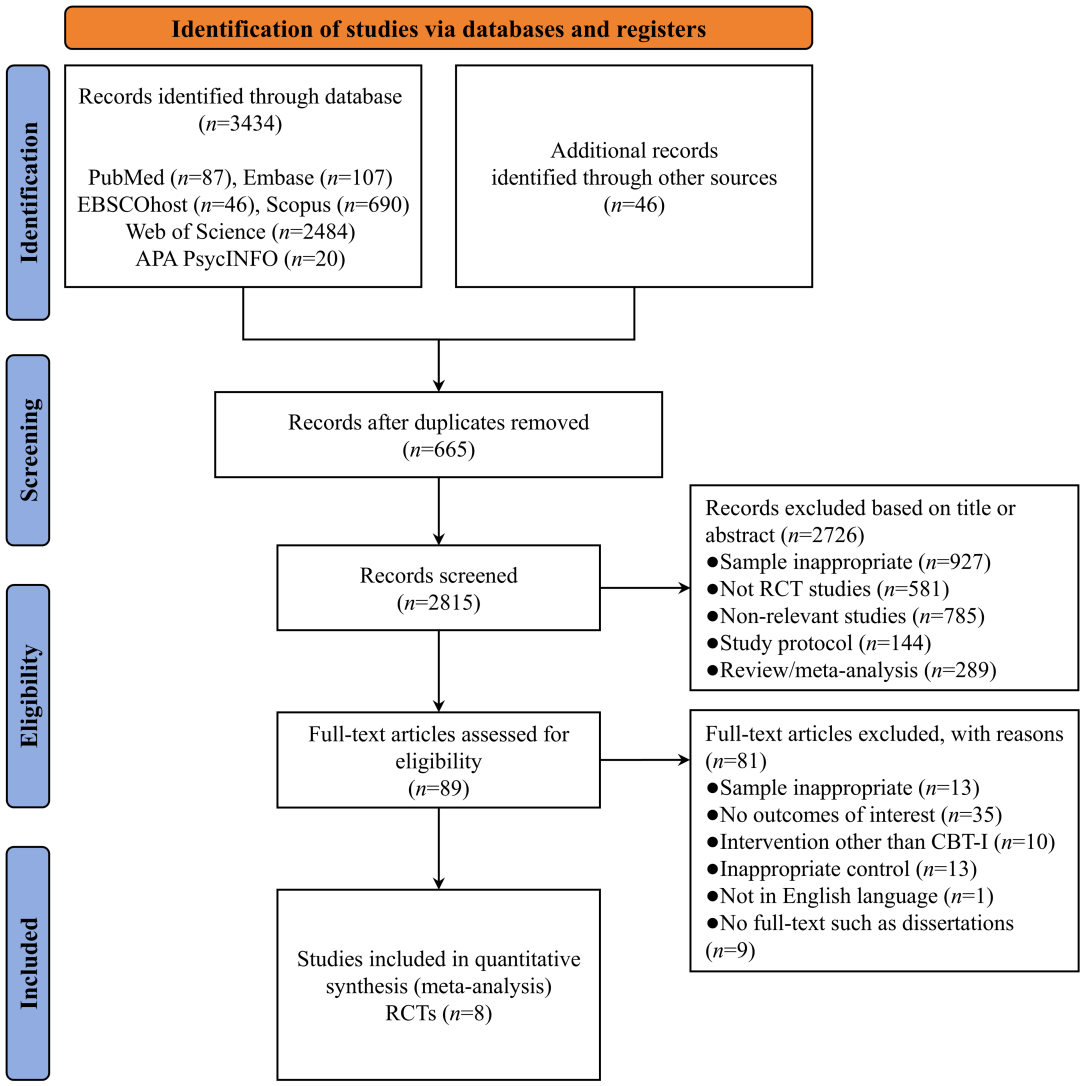
Flowchart of study selection.

A total of 3,480 studies were identified through database searches, including PubMed (*n* = 87), Embase (*n* = 107), EBSCOhost (*n* = 46), Scopus (*n* = 690), Web of Science (*n* = 2,484), APA PsycINFO (*n* = 20), and other sources (*n* = 46). After removing duplicate studies (*n* = 665), the titles and abstracts of 2,815 studies were screened for eligibility, and 2,726 references were eliminated due to samples inappropriate (*n* = 927), not RCT studies (*n* = 581), non-relevant studies (*n* = 785), study protocol (*n* = 144), and review/meta-analysis (*n* = 289). Therefore, 89 studies were subjected to full-text review, 81 of which were deemed ineligible because the sample was inappropriate (*n* = 13), no outcomes of interest (*n* = 35), intervention other than CBT-I (*n* = 10), inappropriate control (*n* = 13), not in English language (*n* = 1), and no full-text such as dissertations (*n* = 9). Finally, 8 studies met the inclusion criteria and were included in the meta-analysis (45–52). A PRISMA flowchart of the literature search is presented in Figure 1.

### 3.2 Study characteristics

Eight full-text RCTs met the inclusion criteria, conducted in various countries: two in China (45, 46), two in the USA (47, 51), two in the Netherlands (48, 49), one in the UK (50), and one in Canada (52). The study populations comprised adolescents with insomnia disorder (6 RCTs) (45–49, 51), adolescents with the highest ranked scores on ISI (1 RCT) (50), and adolescents with persistent postconcussion symptoms (1 RCT) (52). In total, 290 adolescents were assigned to the experimental group, with a mean age ranging from 14.7 to 19.4 years, while 309 were assigned to the control group, with a mean age ranging from 14.7 to 19.7 years.

Most trials referenced accepted diagnostic criteria for insomnia, most commonly the DSM-V (5 RCTs) (45–49), ISI (2 RCTs) (50, 52), and one trial diagnosed insomnia by sleep onset latency and wake after sleep onset (51). Experimental groups comprised group CBT-I (5 RCTs) (45, 46, 48, 50, 52), regular CBT-I and CBT-D (1 RCT) (47), online CBT-I (1 RCT) (49), and SCT (1 RCT) (51). Control groups comprised a wait-list (5 RCTs) (45, 48–50, 52), a no-treatment control (2 RCTs) (46, 51), and sleep hygiene and cognitive behavioral therapy for depression (1 RCT) (47). The main characteristics of the eight RCTs are presented in Table 2.

**Table 2:** Main characteristics of included randomized controlled trials.

Included studies	Population	Age (Mean (SD))	Total/M%	Diagnostic criteria	Intervention	Control	Sleep-related outcomes
Chan (2022) (China) (45)	Adolescents with insomnia	T: 19.4 (2.3) C: 19.7 (2.6)	T: 45/33.3% C: 45/35.6%	DSM-V	Group CBT-I	WL	ISI, PSQI, TST, TIB, SOL, SE, WASO, DBAS
Chan (2021) (China) (46)	Adolescents with insomnia	T: 14.7 (1.8) C: 15.0 (1.7)	T: 121/48.8% C: 121/38.0%	DSM-V	Group CBT-I	NTC	ISI, DBAS, TIB, TST, SOL, SE, WASO
Clarke (2015) (USA) (47)	Adolescents with insomnia or depression	T: 16.5 (1.9) C: 15.9 (1.7)	T: 21/33.3% C: 20/40%	DSISD and DSM-V	Regular CBT-I and CBT-D	Sleep hygiene and CBT-D	ISI, TST, SOL, SE, WASO
de Bruin (2018) (Netherlands) (48)	Adolescents with insomnia	T: 15.6 (1.7) C: 15.9 (1.6)	T: 38/31.6% C: 39/28.2%	DSM-V	Group CBT-I	WL	HSDQ, SE, SOL, TST
de Bruin (2015) (Netherlands) (49)	Adolescents with insomnia	T: NR C: NR	T: 18/NR C: 14/NR	DSM-V	Online CBT-I	WL	HSDQi, CSRQ, TST, TIB, SOL, WASO, SE, SSQ
Egbegi (2021) (UK) (50)	Adolescents with the highest ranked scores on ISI	T: 15.1 (1.13) C: 14.7 (1.17)	T: 21/36% C: 16/36%	Top 50 scorers on ISI	Group CBT-I	WL	ISI, SOL, TSD, SMFQ, SHQ, KSQ
Goodhines (2022) (USA) (51)	Adolescents with sleep disturbance	T: 18.75 (0.62) C: 18.69 (0.90)	T: 14/43% C: 42/38%	Symptom-based diagnosis	SCT	NTC	ISI, Pre-sleep arousal
Tomfohr-Madsen (2020) (Canada) (52)	Adolescents with persistent postconcussion symptoms	T: 15.2 (1.5) C: 14.9 (1.3)	T: 12/25% C: 12/27%	ISI score ≥ 12	Group CBT-I	WL	ISI, SSQ, DBAS, TST, SE, SOL

T, Test group; C, Control group; M%, Percentage of boys; CBT-I, Cognitive behavioral therapy for insomnia; CBT-D, Cognitive behavioral therapy for depression; SCT, Stimulus control treatment; WL, Wait-list; NTC, No-treatment control; DSM-V, Diagnostic and Statistical Manual of Mental Disorders 5; DSISD, Duke Structured Interview for Sleep Disorders; ISI, Insomnia Severity Index; PSQI, Pittsburgh Sleep Quality Index; TST, Total sleep time; TIB, Time in bed; SOL, Sleep onset latency; SE, Sleep efficiency; WASO, Wake after sleep onset; DBAS, Dysfunctional Belief Attitude Scale; HSDQ, Holland Sleep Disorder Questionnaire; HSDQi, Insomnia Scale Holland Sleep Disorder Questionnaire; CSRQ, Chronic Sleep Reduction Questionnaire; SSQ, Subjective sleep quality; TSD, Total sleep duration; SMFQ, Short Mood and Feelings Questionnaire; SHQ, Sleep Hygiene Questionnaire; KSQ, Knowledge of Sleep Questionnaire.

### 3.3 Risk of bias

**Figure 2:**
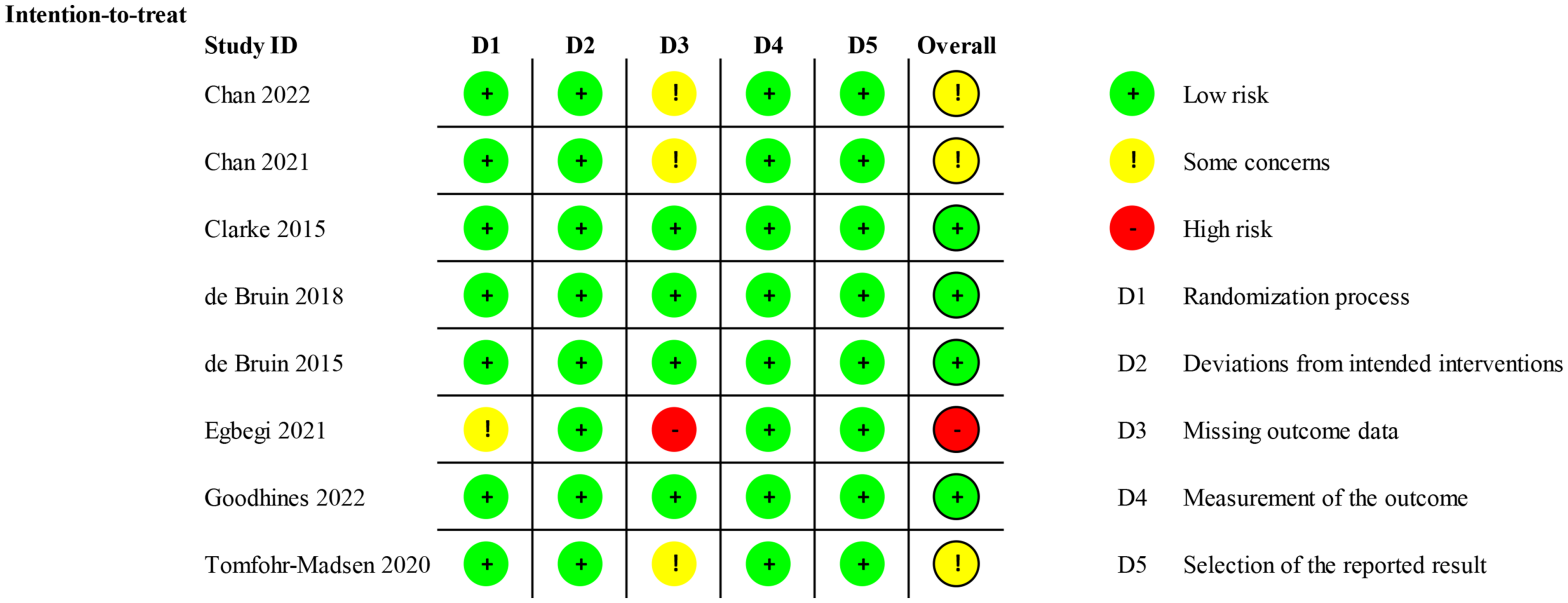
Risk of bias graph.

**Figure 3:**
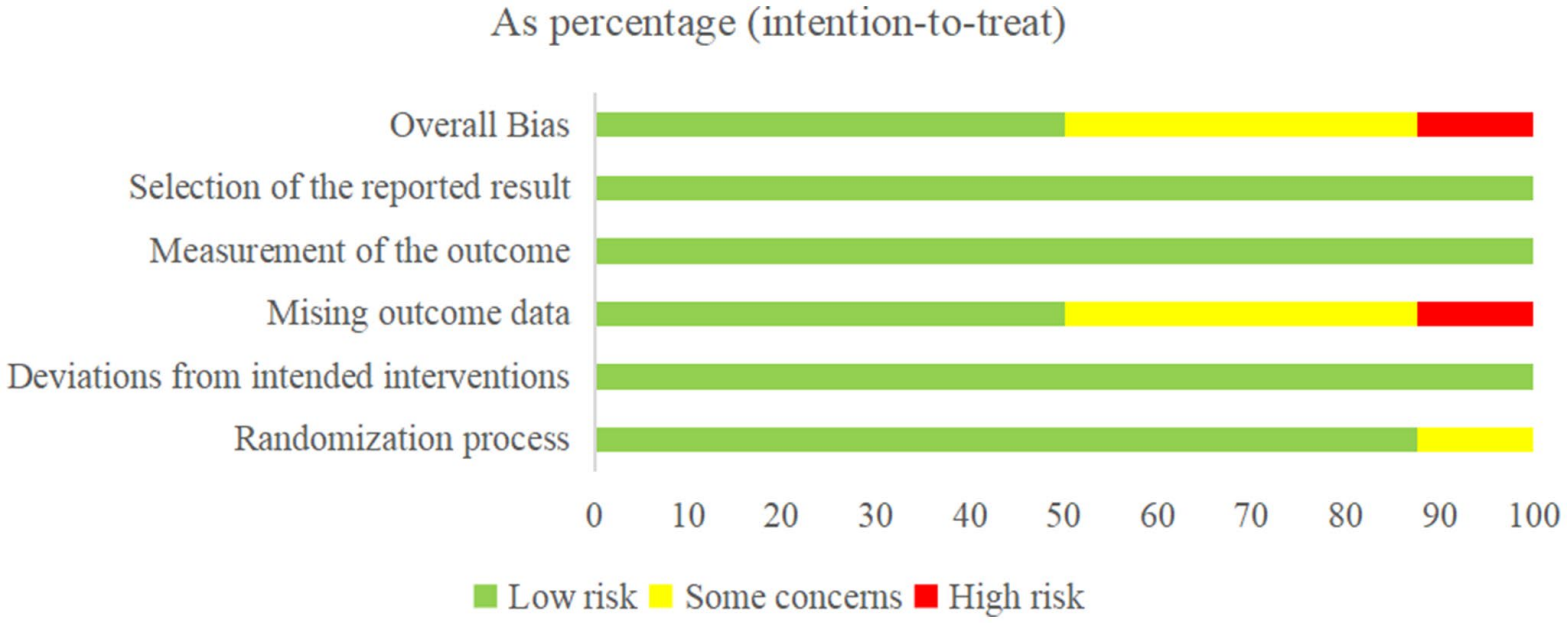
Risk of bias summary.

Seven trials showed a low risk of bias in the randomization process (45–49, 51, 52), and one trial was assessed as having some concerns owing to the random allocation sequence (50). For deviations from intended interventions, all trials were considered low risk because there were no deviations from intended interventions that arose because of the experimental context. For missing outcome data, four trials were of low risk because the data for the outcome were available for all or nearly all randomized participants (47–49, 51). Three trials had some concerns (45, 46, 52) and one had high risk because participant data were incomplete and there was no evidence that the result was not biased by missing outcome data (50). The measurement of the outcome and selection of the reported result were low risk because all trials used appropriate methods to measure outcomes, and all measurements and data analyses were available and none of the trials reported results selectively. The overall risk was low risk in four trials (47–49, 51), with some concerns in three trials (45, 46, 52), and high risk in one trial (50). The Cochrane risk-of-bias assessment is presented in Figures 2, 3.

### 3.4 Meta-analysis

**Figure 4:**
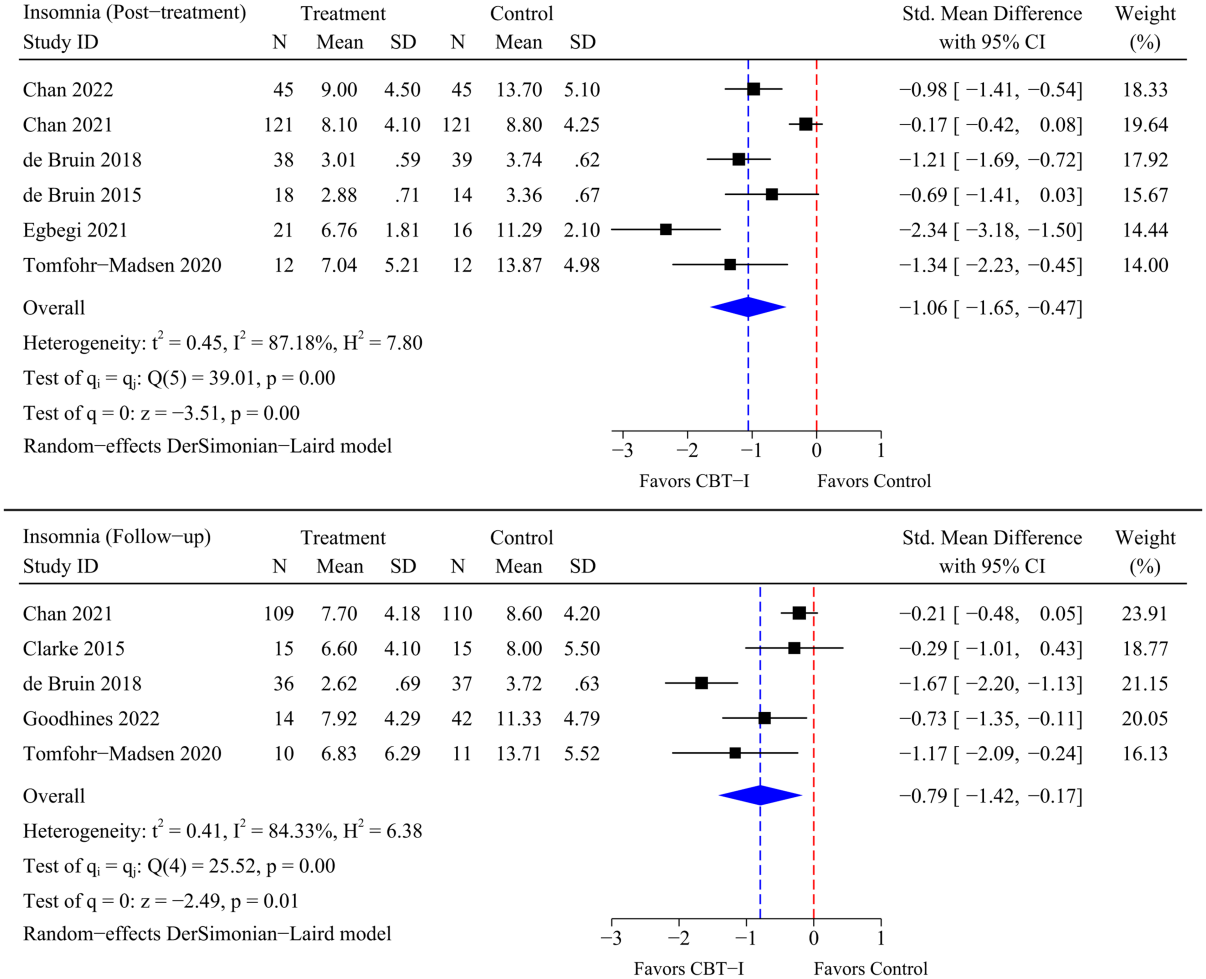
Meta-analysis of the efficacy of CBT-I on insomnia.

**Figure 5 fig5:**
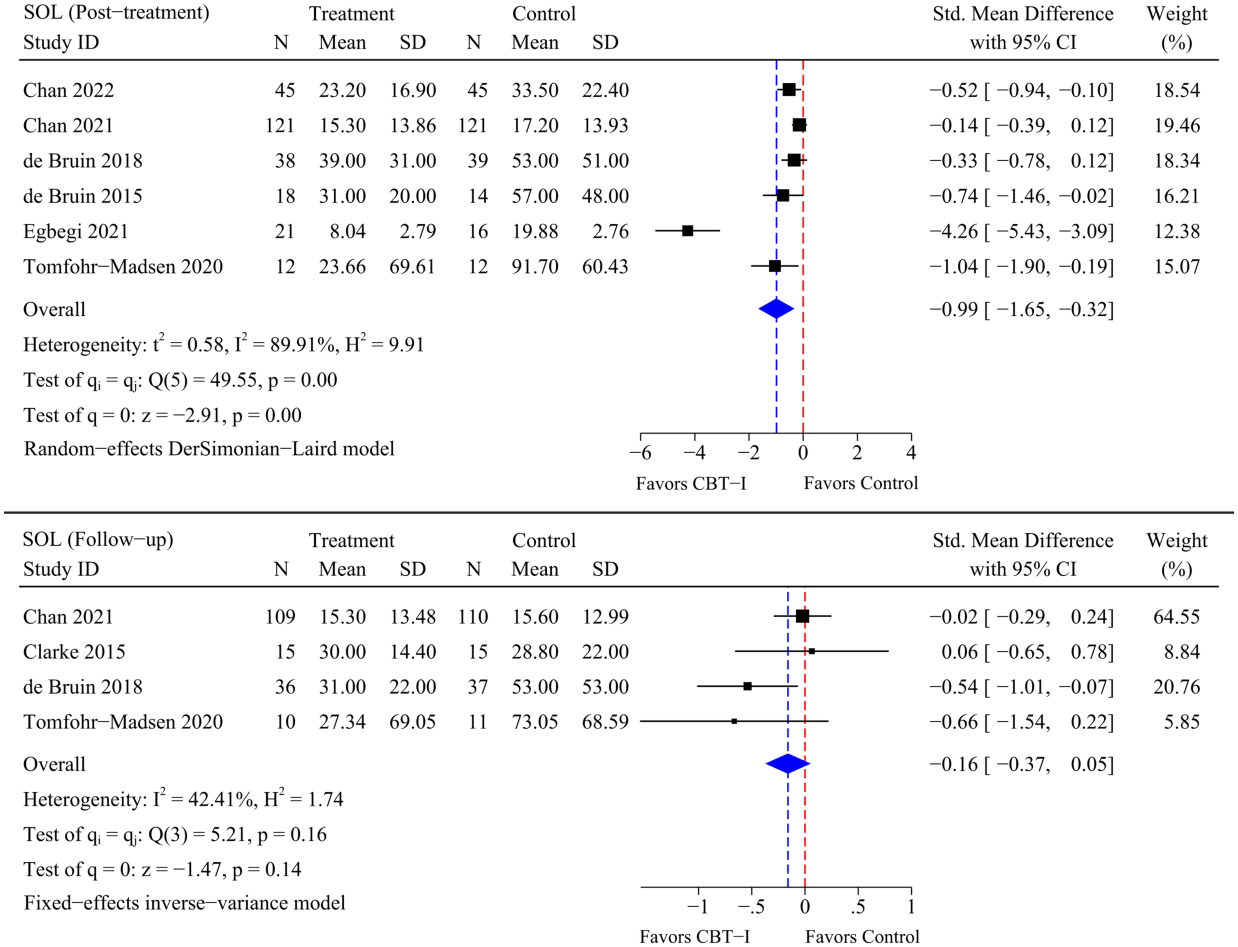
Meta-analysis of the efficacy of CBT-I on SOL.

**Figure 6 fig6:**
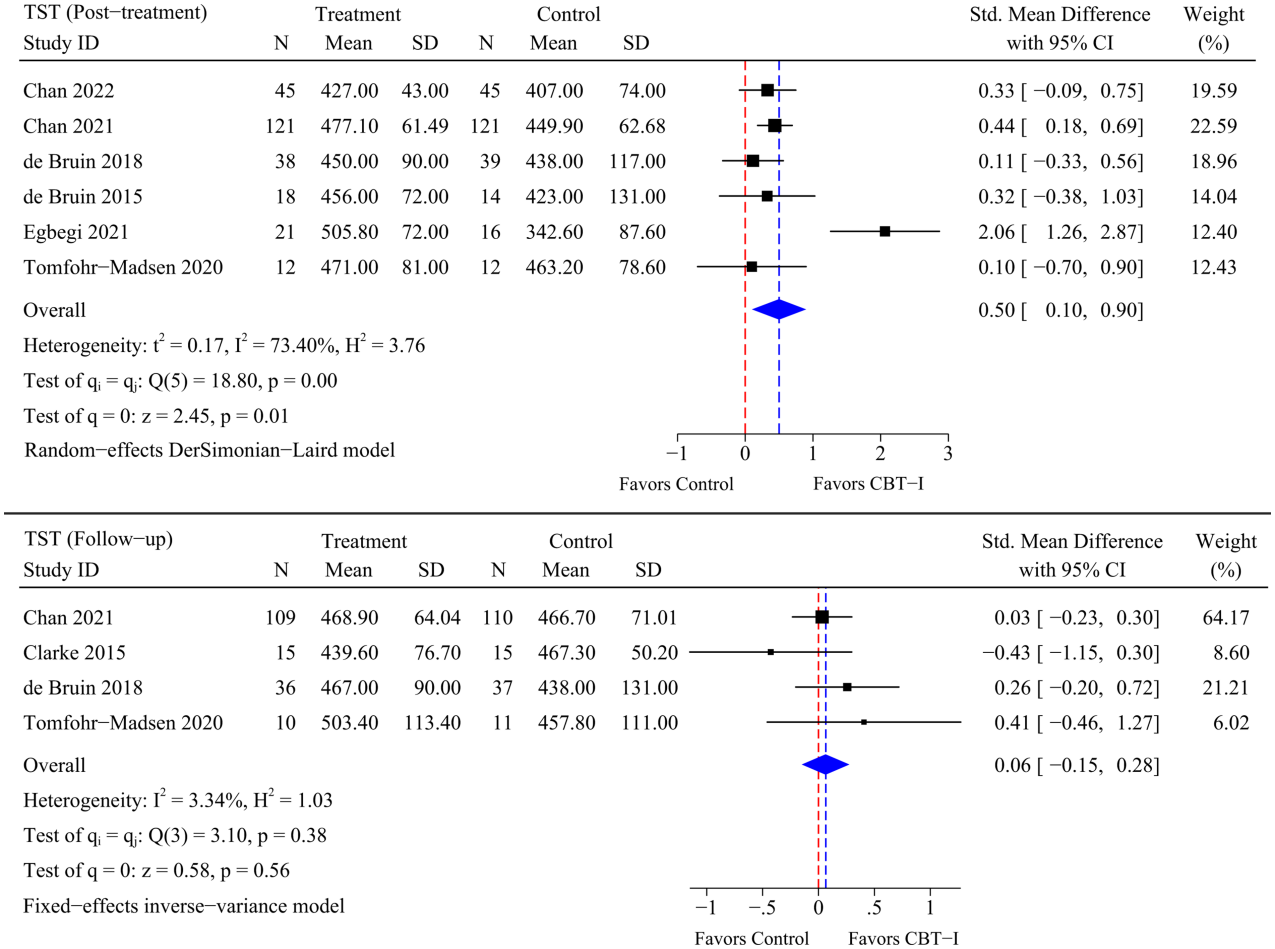
Meta-analysis of the efficacy of CBT-I on TST.

A total of eight RCTs were included in the meta-analysis, and heterogeneity was examined using the Chi-square test based on *Q*-test and *I^2^* statistics. Marked and statistically significant improvements in insomnia (SMD = −1.06; 95% CI -1.65 to −0.47; *p* < 0.01), SOL (SMD = −0.99; 95% CI -1.65 to −0.32; *p* < 0.01), TST (SMD = 0.50; 95% CI 0.10 to 0.90; *p* = 0.01), and SE (SMD = 0.57; 95% CI 0.26 to 0.87; *p* < 0.01) were observed at post-treatment time point following CBT-I. At follow-up time point, a statistically significant improvement in insomnia (SMD = −0.79; 95% CI -1.42 to −0.17; *p* = 0.01) was observed following CBT-I. Although there were no significant improvements in the remaining sleep-related outcomes, statistical significance was borderline as there were fewer RCTs available for the meta-analysis at follow-up time point. The results of the meta-analysis for each outcome are presented in Figures 4–7.

**Figure 7:**
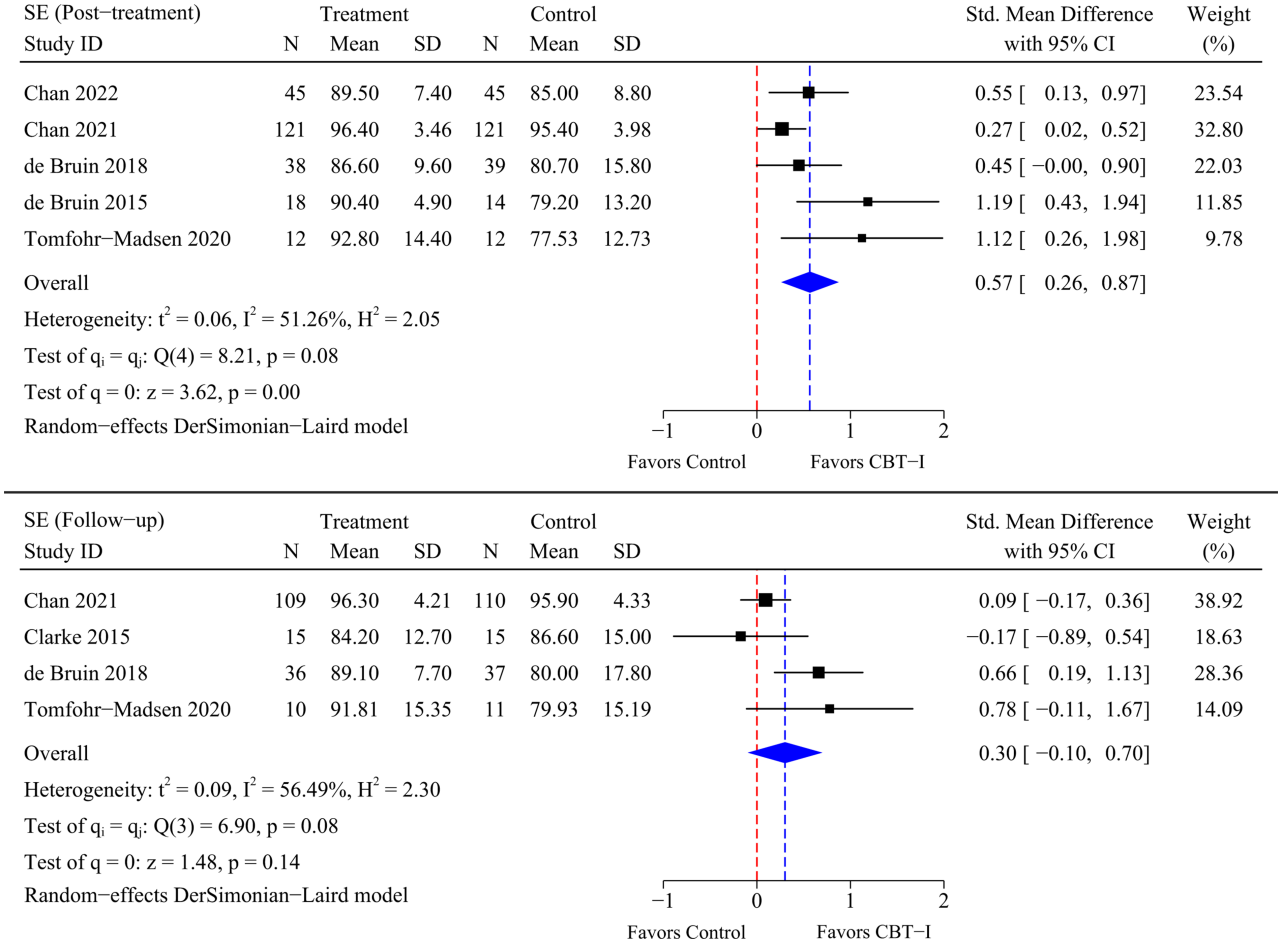
Meta-analysis of the efficacy of CBT-I on SE.

### 3.5 Sensitivity analysis

The results of the sensitivity analysis indicated that the pooled results for insomnia, SOL, and SE remained stable at post-treatment time point after excluding individual studies, indicating that these results were insensitive to study selection. However, the pooled results of TST were statistically changed and sensitive to study selection when the Chan et al. (46) study was excluded (SMD = 0.54; 95% CI -0.03 to 1.11; *p* > 0.05). At follow-up time point, the pooled results for insomnia, TST, and SE were more insensitive to study selection. However, the pooled results of SOL were statistically changed and sensitive to study selection when the Chan et al. (46) study was excluded (SMD = −0.41; 95% CI -0.77 to −0.05; *p* < 0.05).

The differences mentioned above may be attributed to the larger sample size of the excluded study, which was significantly weighted in the meta-analysis and therefore had a greater impact on the pooled results. In addition, statistical significance was generally borderline due to the limited number of trials that met the inclusion criteria, resulting in the pooled results for some sleep-related outcomes being sensitive to study selection. The results of the sensitivity analysis for each outcome are presented in Table 3.

**Table 3:** Sensitivity analysis for outcomes by omitting individual studies.

Outcomes	Time points	Study omitted	SMD	95% CI
Insomnia	Post-treatment	Chan (2022) (45)	−1.10	(−1.85, −0.35)
		Chan (2021) (46)	−1.25	(−1.69, −0.80)
		de Bruin (2018) (48)	−1.04	(−1.73, −0.34)
		de Bruin (2015) (49)	−1.14	(−1.83, −0.45)
		Egbegi (2021) (50)	−0.83	(−1.35, −0.31)
		Tomfohr-Madsen (2020) (52)	−1.02	(−1.67, −0.37)
	Follow-up	Chan (2021) (46)	−0.98	(−1.62, −0.34)
		Clarke (2015) (47)	−0.92	(−1.68, −0.15)
		de Bruin (2018) (48)	−0.47	(−0.86, −0.87)
		Goodhines (2022) (51)	−0.81	(−1.61, −0.16)
		Tomfohr-Madsen (2020) (52)	−0.72	(−1.43, −0.01)
Sleep onset latency	Post-treatment	Chan (2022) (45)	−1.14	(−2.01, −0.27)
		Chan (2021) (46)	−1.24	(−2.12, −0.35)
		de Bruin (2018) (48)	−1.18	(−2.03, −0.32)
		de Bruin (2015) (49)	−1.05	(−1.81, −0.28)
		Egbegi (2021) (50)	−0.41	(−0.69, −0.14)
		Tomfohr-Madsen (2020) (52)	−0.98	(−1.71, −0.24)
	Follow-up	Chan (2021) (46)	−0.41	(−0.77, −0.05)
		Clarke (2015) (47)	−0.18	(−0.40, 0.04)
		de Bruin (2018) (48)	−0.06	(−0.30, 0.18)
		Tomfohr-Madsen (2020) (52)	−0.13	(−0.35, 0.09)
Total sleep time	Post-treatment	Chan (2022) (45)	0.55	(0.04, 1.07)
		Chan (2021) (46)	0.54	(−0.03, 1.11)
		de Bruin (2018) (48)	0.59	(0.12, 1.07)
		de Bruin (2015) (49)	0.53	(0.07, 0.99)
		Egbegi (2021) (50)	0.34	(0.15, 0.52)
		Tomfohr-Madsen (2020) (52)	0.56	(0.12, 1.00)
	Follow-up	Chan (2021) (46)	0.12	(−0.24, 0.47)
		Clarke (2015) (47)	0.11	(−0.11, 0.33)
		de Bruin (2018) (48)	0.01	(−0.23, 0.25)
		Tomfohr-Madsen (2020) (52)	0.04	(−0.18, 0.26)
Sleep efficiency	Post-treatment	Chan (2022) (45)	0.62	(0.20, 1.04)
		Chan (2021) (46)	0.69	(0.36, 1.02)
		de Bruin (2018) (48)	0.65	(0.23, 1.06)
		de Bruin (2015) (49)	0.45	(0.19, 0.70)
		Tomfohr-Madsen (2020) (52)	0.49	(0.20, 0.79)
	Follow-up	Chan (2021) (46)	0.43	(−0.13, 0.99)
		Clarke (2015) (47)	0.42	(−0.05, 0.89)
		de Bruin (2018) (48)	0.14	(−0.23, 0.51)
		Tomfohr-Madsen (2020) (52)	0.22	(−0.21, 0.65)

SMD, Standardized Mean Difference; CI, Confidence Interval.

## 4 Discussion

Addressing sleep disorders in adolescents through effective psychological interventions, such as CBT-I, is crucial for their physical and mental health, as these disorders are associated with various adverse outcomes, especially suicidal ideation (53), emotional regulation disorder (54), and substance abuse (55). The objective of this systematic review and meta-analysis was to evaluate the overall efficacy of CBT-I in treating insomnia in adolescents, and to examine the efficacy of CBT-I on different sleep-related outcomes in this population. The pooled results of the meta-analysis indicated that CBT-I, at post-treatment time point, effectively improved insomnia (SMD = −1.06; 95% CI -1.65 to −0.47; *p* < 0.01) and some sleep-related outcomes, including SOL (SMD = −0.99; 95% CI -1.65 to −0.32; *p* < 0.01), TST (SMD = 0.50; 95% CI 0.10 to 0.90; *p* = 0.01), and SE (SMD = 0.57; 95% CI 0.26 to 0.87; *p* < 0.01) in adolescents. At follow-up time point, although SOL, TST, and SE failed to improve, CBT-I still showed an improvement effect on insomnia in adolescents (SMD = −0.79; 95% CI -1.42 to −0.17; *p* = 0.01).

Considering that circadian rhythm delay is prominent in adolescence and can be easily confused with insomnia, this study adhered strictly to pre-established criteria during the study selection process to eliminate the potential influence of other factors on the research results. Specifically, participants in five trials were diagnosed with insomnia using instruments such as the DSM-V (45–49). Additionally, they were required to engage in structured screening interviews (including assessments of sleep patterns and sleep disorders) with clinical professionals or related experts, to ascertain their eligibility for an insomnia diagnosis. One trial utilized the Morningness-Eveningness Questionnaire to control for potential circadian rhythm/phase disorders (51). Furthermore, in terms of participant characteristics, all trials excluded participants who were using sleep medications. However, two trials diagnosed insomnia solely based on ISI without considering the impact of circadian rhythm delay (50, 52). The results of the sensitivity analysis indicated that excluding these trials did not significantly affect the pooled results of the outcomes, suggesting that the potential influence of circadian rhythm delay on the findings of this study can be ruled out.

In fact, the mechanisms by which CBT-I improves insomnia primarily rely on behavioral and cognitive strategies. Behavioral strategies for insomnia aim to modify sleep-incompatible behaviors in the bedroom and consolidate sleep architecture (56, 57). Cognitive strategies target erroneous beliefs maintaining insomnia (56). For instance, in terms of behavioral strategies, sleep restriction therapy improves insomnia primarily by reducing the tendency to remain in bed while awake (58). Specifically, by restricting time in bed (TIB), sleep restriction therapy curtails sleep opportunity, resulting in partial sleep deprivation, which contributes to an increase in sleep need, thereby enhancing sleep efficiency and quality (59). Stimulus control therapy can regulate the relationship between TIB and bedtime through sleep instructions, such as “Lie down, intending to go to sleep only when you are sleepy,” thereby reinforcing sleep behaviors while inhibiting non-sleep behaviors (60–62). In terms of cognitive strategies, cognitive therapy is able to identify, challenge, and replace dysfunctional beliefs and attitudes about sleep and insomnia, such as “If I cannot sleep all the time, I may crash” (63). This intervention may help individuals fall asleep faster, and improve their sleep quality (63, 64). Furthermore, insomniacs have greater activation in cognitive, emotional, and cortical domains, and CBT-I can decrease such activation by diverting attention from sleep difficulties, thereby alleviating sleep disturbances in these patients (65, 66). Finally, insomnia is usually associated with hyperactivation of the hypothalamic–pituitary–adrenal (HPA) axis (2, 67, 68), and impaired heart rate variability (HRV) (69, 70). Evidence suggests that CBT-I helps to suppress hyperactivation of the HPA axis, reduce hyperarousal, and improve HRV during sleep (71, 72). It is important to note that the aforementioned physiological changes represent correlational rather than causal relationships, and are primarily based on data from studies of adult populations. For adolescents whose brains are still developing, these physiological changes may exhibit different relevance and applicability. Therefore, the specific physiological mechanisms by which CBT-I improves insomnia in adolescents require further investigation and validation to comprehensively clarify the efficacy of this therapy in this population.

The pooled results of the current systematic review and meta-analysis indicated that CBT-I was effective in improving insomnia in adolescents compared with the control group, both at post-treatment time point and at follow-up time point. For some sleep-related outcomes, including SOL, TST, and SE, none of the CBT-I group showed a statistically significant difference in therapeutic efficacy compared with the control group at follow-up time point. It has been hypothesized that this may be due to the fact that CBT-I strategies implemented throughout the course of therapy were not used by adolescent patients any more to manage sleep disorders over time, leading to a “sharp decline” in the improvement of sleep-related outcomes at follow-up time point (73). This “sharp decline” suggests that adolescent adherence to CBT-I strategies may decrease over time. Effectively managing this decline in adherence and maximizing the long-term efficacy of CBT-I on insomnia and sleep-related outcomes in adolescents are critical issues that warrant further investigation. Furthermore, the confidence intervals for some pooled results were close to the margin of statistical significance, which implies that the pooled results may vary in the case of a change in the study selection or in certain conditions. This suggests the sample size and experimental design should be further expanded and optimized, in order to assess the long-term effects of CBT-I on these outcomes more accurately.

In summary, the findings of this study suggest that CBT-I was effective in improving insomnia in adolescents and some sleep-related outcomes, including SOL, TST, and SE. Adolescents are at a critical stage in healthy physical and mental development, during which they are often exposed to physical, psychological and, social stressors. The cumulative effect of these stressors may increase the risk of insomnia in adolescents. CBT-I was characterized by low risk and high therapeutic benefits and could serve as alternative or adjuvant approaches to medication for the treatment of insomnia. In a direct comparison, CBT-I has been shown to be more effective than medication in managing insomnia, with effects lasting for at least 6 months of follow-up (74). Therefore, considering the advantages in terms of safety and efficacy, CBT-I should be the preferred intervention for the treatment of insomnia in adolescents. Appropriate medication can be adopted in accordance with specific conditions to further enhance the therapeutic effects, and the improvement in sleep quality and related disorders can be maximized though this comprehensive treatment in adolescent patients.

## 5 Limitations

The findings of the current systematic review and meta-analysis must be interpreted in the context of limitations. First, due to the limited number of trials that met the inclusion criteria, some additional sleep-related outcomes, including TIB, WASO, and SSQ were not covered in this study. Second, the follow-up time point range was limited to 6 months or less due to the different follow-up time points among the included trials. The therapeutic efficacy of CBT-I for insomnia in adolescents beyond 6 months was not investigated. Therefore, future research should be encouraged to increase sample sizes and extend follow-up periods based on these findings, in order to explore the more comprehensive and long-term effects of CBT-I on sleep-related outcomes in adolescents. It is important to note that this study did not conduct subgroup analyses based on demographic data, which means that the specific efficacy of CBT-I on sleep-related outcomes in adolescents remains unconfirmed. Addressing this gap will contribute to a more nuanced understanding of the differential benefits of CBT-I in this field, which could further facilitate the development of personalized CBT-I strategies. Finally, the confidence intervals for some of the pooled results were close to the margin of statistical significance. This suggests that the pooled results may vary in the case of a change in the study selection or in certain conditions, and these findings should be interpreted cautiously.

## 6 Conclusion

The objective of this systematic review and meta-analysis was to evaluate the overall efficacy of CBT-I in treating insomnia in adolescents, and to examine the efficacy of CBT-I on different sleep-related outcomes in this population. CBT-I was effective in improving insomnia in adolescents and some sleep-related outcomes, including SOL, TST, and SE. Moreover, CBT-I was characterized by low risk and high therapeutic benefits and could serve as alternative or adjuvant approaches to medication for the treatment of insomnia. Therefore, considering the advantages in terms of safety and efficacy, CBT-I should be the preferred intervention for the treatment of insomnia in adolescents.

## Data Availability

The original contributions presented in the study are included in the article/supplementary material, further inquiries can be directed to the corresponding author.
